# Medicine

**Published:** 1892-12

**Authors:** J. E. Shaw


### Progress of the Medical Sciences.

### MEDICINE.

The discussion on phagocytosis and immunity at the Pathological Society, Sidney Martin's Goulstonian lectures, Sims Woodhead's address on bacteriology, and the discussion on immunity at Nottingham this year, show the increasing attention which is being paid to this department of medical science. Interesting as it would be to discuss the different views upon phagocytosis and immunity, their consideration belongs rather to pathology than to medicine, so it seems more appropriate here simply to recount some recent gains to clinical medicine through bacteriological research. First, however, we would note, as being of general interest, (1) the vastly greater bactericidal power of blood-serum compared to that of blood-plasma; (2) the superiority of the immediate benefit obtained from treatment by immunised serum over the slowly developed immunity produced by inoculation; and (3) the complete analogy of some toxalbumins not produced by bacteria (*e.g.* abrin) with those derived from pathogenic micro-organisms. Ehrlich has shown [1] that susceptible animals may be made immune against these proteids of non-bacterial origin, and that the blood of such immune animals contains an antitoxine to which this immunity is due.

Amidst the deluge of literature which has appeared upon the subject of cholera recently, something has been added to our knowledge of the bearing of the bacteriology of the disease upon its cure. Protective inoculations of animals against cholera have been successfully made by many experimenters. Brieger and Wasserman,[2] G. Klemperer,[3] and L. Vincenzi [4] record their independent success in conferring immunity by injecting differently prepared cultures of the cholera vibrio. More interesting to us, however, is the report by Mr. E. H. Hankin [5] of his protective inoculation of himself in M. Haffkine's laboratory in Paris. An abnormally virulent

[1] *Deutsch. med. Woch.*, 1891, No. 44.
[2] *Deutsch. med. Woch.*, Aug. 4, 1892. [3] *Berlin. klin. Woch.*, Aug. 8, 1892.
[4] *Arch. delle Scienze Med.*, vol. xxi., fasc. 3.
[5] *Brit. Med. Journ.*, Sept. 10, 1892.

culture—*virus fixe* or *exalté*—as well as an attenuated culture, having been obtained, Mr. Hankin, with seven others, were "vaccinated," first with the attenuated, and a week later with the virulent culture, causing phenomena described by Hankin, and rendering these inoculated persons insusceptible to any further influence of the virus administered in the same manner. More recently we have seen it stated that Mr. Hankin has swallowed some culture of the cholera vibrio with impunity, a result unlike that obtained in Berlin, where the accidental swallowing of such by an "unvaccinated" person led to a prompt attack of cholera, with abundance of the "comma bacilli" in the intestinal evacuations. As Haffkine's method can be practised in human beings with perfect safety, and as it certainly produces immunity against cholera in animals so widely different as pigeons and guinea-pigs, results already obtained made it reasonable to believe that it produces similar effects on human beings.

The cholera vibrio flourishes only in alkaline media, and cholera can be caused in the guinea-pig by oral administration of the vibrio, only if the gastric juice be neutralised with soda and the peristaltic action of the intestines diminished by opium. The modern medicinal treatment of cholera therefore consists in avoiding the administration of opium and the plentiful administration of acids in diluent drinks. Ferran recommends[1] lactic acid in lemonade, while Cantani[2] prefers the administration of very copious enemata (so that the solution shall pass backward through the ileo-cæcal valve) of tannic acid, under which treatment he says nearly every case recovers.

The specificity of the contagium of typhoid fever has again been seriously called in question. Dr. Gabriel Vallet[3] maintains that Eberth's bacillus is only a modification in its passage through the body of the bacillus coli communis. Frequently in typhoid stools this latter organism is found alone, and Eberth's bacillus, when present, is found between the tenth and twentieth day, when Peyer's patches have commenced to ulcerate, and time has permitted the bacillus coli to undergo within them the same modifications it is supposed to do in the spleen, being transformed there into Eberth's bacillus. The two organisms differ very little in

1 *Compt. Rend. de l'Acad. des Sciences*, cxv. 101, 1892.

2 *Berlin. klin. Woch.*, Sept. 12, 1892.

3 *Le bacillus coli communis dans ses rapports avec le bacille d'Eberth et l'étiologie de la Fièvre Typhoide.*

their morphological or other characters, and when tested on *animals* their pathogenic properties seem identical. Dr. Vallet has therefore come to the conclusion that in typhoid, as has been described in diphtheria, there are a pathogenic and a non-pathogenic form of the same organism. Bacillus coli communis propagated in fæcal solutions becomes much more virulent. It is important, therefore, to prevent accumulation and fermentation of fæcal matters; for possibly Murchison was right in his view that typhoid can arise *de novo* by such fermentation. Dr. Vallet cannot be said to have actually proved his theory, and other observers differ from him in their views as to the difference of the two bacilli. Theobald Smith [1] describes distinguishing chemical differences, and Luksch [2] distinctive microscopical appearances and staining properties between the two micro-organisms. Stern [3] records experiments showing that immunity against typhoid can be obtained by injection of blood-serum derived from persons convalescent from the disease. Bruschettini [4] has also obtained complete immunity in animals by employing cultures of Eberth's bacillus, obtained from the spleen of a typhoid patient, administered subcutaneously. Serum of rabbits thus immunised has a much greater bactericidal power in relation to the typhoid bacillus than that of normal animals, and possesses also a very marked antitoxic action to the virus of the typhoid bacillus.

Dr. T. M. Prudden records [5] later experiments confirming those by Straus, Vissman, and his own earlier investigation, which show that injection of dead tubercular bacilli causes the development of new growths anatomically similar to ordinary "tubercles," but that no caseation takes place in them, nor does any constitutional infection arise from them as they contain no living bacilli. Cheesy degeneration is apparently due to some metabolic product of the bacillus wholly distinct from the cell-stimulating bacterial protein, while there is still another agency of a toxic nature to which many of the graver systemic effects of the tubercular infection are due: emaciation however is observed after the injection of dead bacilli. In these artificial "tubercles" the injection of tuberculin does not cause caseation; for, as Maffucci [6] has shown, the active substance of tubercle

[1] *Central. für Bakt.*, Mar. 19, 1892. [2] *Ibid*, Sept. 28, 1892.

[3] *Deutsch. med. Woch.*, Sept. 15, 1892.

[4] *Rif. Med.*, Aug. 9, 1892. [5] *N.Y. Med. Journ.*, Sept. 10, 1892.

[6] *Rif. Med.*, Nov. 24, 1891.

culture is retained in the bacilli, not in the liquid serum. At the International Congress at Berlin, Koch declared the specific difference between avian and human tuberculosis; more recently, Gamaleïa and Straus [1] have indisputably distinguished between them. French investigations upon tuberculosis have been made upon the avian species, and their results therefore are not certainly true as to mankind. Nevertheless Héricourt [2] adheres to his view, that it is by successive vaccinations of avian tubercle that dogs are rendered immune to human tuberculosis, to which they are naturally prone. This immunised serum he considers a valuable restorative in tuberculosis in man.

In tetanus we may consider most completely to have advanced towards cure by bacteriological methods. The antitoxine can be precipitated by alcohol from the blood of dogs or rabbits strongly immunised against tetanus, dried *in vacuo*, and kept until required to be used. From 15 to 25 centigrammes, dissolved in a little water, are injected once or twice daily. About four or five injections are usually required to effect a cure. This plan, which will conveniently supersede treatment by immunised serum, has been employed in instances of tetanus in man, and successful cases have been recorded by Pacini, Nicoladoni, Schwarz, Finotti, Bruschettini, Taruffi, and Casali, and possibly by others also, the curative substance being obtained from Tizzoni of Bologna.

Bruschettini [3] and Taruffi [4] point out that the toxine of tetanus is eliminated in the urine of the animal suffering from the disease; the efficacy of the antitoxine therefore can be gauged by the degree of inability of the urine from the tetanic animal under treatment to produce tetanus when injected into another animal not previously rendered immune.

Tizzoni and Cattani report [5] that if both parents have been immunised to tetanus, their offspring are hereditarily immune; but immunity is also often, if not always, to be obtained through the milk of an immune foster-mother, as has been elsewhere proved.

Koplik has again proved,[6] by elaborate investigation, that the certain diagnosis of diphtheria is impossible clini-

[1] *Archiv. de Méd. Expér.*

[2] *Archiv. gén. de Méd.*, April, 1892, and *Gaz. méd. de Paris*, Oct. 22, 1892.

[3] *Rif. Med.*, April 11 and July 29, 1892. [4] *Ibid.*, April 21, 1892.

[5] *Ibid.*, April 26, 1892.

[6] *N.Y. Med. Journ.*, Aug. 27, 1892.

cally, the presence of membrane and other signs, which used to be relied upon, being of no critical value. Baginsky also[1] has come to the same conclusion. Both authorities agree that "diphtheritic" membrane may be produced by Löffler's bacillus, by the pseudo-bacillus (of Hofman), or by strepto-cocci and staphylococci. True diphtheria may equally be present in the absence of membrane, and such cases—diphtheria *sine membranâ*—are the more insidiously dangerous on account of their real nature not being suspected. Woodhead, in his address on bacteriology at Nottingham,[2] commenting upon the same point, sensibly urges that, as it is impossible for the majority of practitioners to do more than make the preliminary steps of the bacteriological investigation, which is necessary to make a diagnosis, some central authority, such as the Association for the Advancement of Medical Research, should, for a suitable fee, undertake the investigation of specimens sent them.

Notwithstanding Behring's experiments, authorities more recently are not sanguine that protective or curative inoculation can be successfully performed for diphtheria. Only partial immunity has been hitherto obtained, and the ulterior effect of the toxic albumose renders its administration not devoid of risk. In this Koplik, Woodhead, and Sidney Martin [3] are practically agreed.

Hughes and Carter of Philadelphia,[4] in reporting a case of pneumonia treated by transfusion of blood from convalescent case, quote previous experience in the cure of pneumonia by the immune serum of convalescents. Mosny, Klemperer (six cases), Neisser (three cases), Redner (twenty cases), and Janson (several cases) show that the method first practised by G. and F. Klemperer has met with widely extended success. It would appear that the substance conferring immunity is not identical with the antitoxin, but that the former generates the latter; neither body has been isolated. After an attack of pneumonia the serum retains its immunifying power for about three months. The quantity of serum employed has varied considerably in the hands of different experimenters; the authors think that not less than 50 c.c. should be used.

The bacteriological cause of influenza may now be con-

[1] *Archiv. für Kind.*, xiii. [2] *Brit. Med. Journ.*, Aug. 6, 1892.

[3] Goulstonian lectures, 1892. [4] *Therap. Gaz.*, Oct. 1892.

sidered as settled. Barreggi,[1] Bruschettini,[2] and Cornil and Chantemesse[3] record finding the bacillus already described by Pfeiffer and Canon. Pfeiffer himself and Beck[4] make further report to the effect that the bacillus is found in the bronchial secretion, not in the blood, so that apparently the primary morbid process of influenza is accomplished within the bronchial territory. In no case of influenza have the authors failed to find these bacilli, nor were they ever present when influenza could be excluded. Clinical experience would lead us to believe that the duration of immunity must be extremely brief.

J. E. SHAW.

### SURGERY.

[1] *Gazz. Med. Lomb.*, Jan. 16, 1892. [2] *Rif. Med.*, Jan. 29, 1892.

[3] *Sem. Méd.*, Feb. 10, 1892.

[4] *Deutsch. med. Woch.*, May 26, 1892.

